# Tuberculosis among Health Workers—A Secondary Data Analysis of German Social Accident Insurance Data from 2002–2017

**DOI:** 10.3390/ijerph17051564

**Published:** 2020-02-28

**Authors:** Jan Felix Kersten, Albert Nienhaus, Stephanie Schneider, Anja Schablon

**Affiliations:** 1 Competence Centre for Epidemiology and Health Services Research for Healthcare Professionals (CVcare), University Medical Centre Hamburg-Eppendorf (UKE), 20246 Hamburg, Germany; a.nienhaus@uke.de (A.N.); a.schablon@uke.de (A.S.); 2 Department of Occupational Medicine, Public Health and Hazardous Substances, Institution for Statutory Accident Insurance and Prevention in the Health and Welfare Services, 22089 Hamburg, Germany; 3 Deutsche Gesetzliche Unfallversicherung (DGUV), 10117 Berlin, Germany; stephanie.schneider@dguv.de

**Keywords:** occupational disease, tuberculosis, health workers, secondary data analysis, tuberculosis latency, comparison of TB recognitions of insurance carriers

## Abstract

Tuberculosis (TB) is the most common cause of fatal infections worldwide. Recent TB figures in Europe indicate that 30 people were infected with tuberculosis each hour in 2017. Healthcare workers are at particular risk of being infected through patient contact. TB is the second most common occupational infectious disease among German healthcare workers. Routine data from the German Social Accident Insurance were used to examine trends in occupational TB diseases. We analyzed annual cross-sectional data for the years 2002 to 2017. The data underwent descriptive analysis. A total of 4653 TB cases were recognized as occupational diseases (OD) in the period under study. In 2002, 60 TB cases were recognized as OD No. 3101, i.e., transmissions from person to person. Since 2013, the level has settled at around 500 recognized cases per year. This is around eight times the number of cases compared to 2002. The following three groups collectively accounted for the largest share of TB cases (88.5%): nurses (including geriatric nurses), other healthcare employees, and physicians. The upward trend in the number of TB cases recognized as occupational diseases is probably due to improvements in diagnostic tests used to diagnose TB infections. TB in health and welfare workers remains an important issue in the health and welfare sector in Germany, partly due to the long latency period between potential exposure to infectious patients or materials and the recognition of the latent tuberculosis infection (LTBI) or active TB as OD.

## 1. Introduction

A range of Western European countries have been considered low-incidence countries for tuberculosis (TB) for several years. However, tuberculosis is the infectious disease with the highest mortality figures worldwide [1,2,3]. Recent figures from Europe indicate that around 30 people an hour contract TB today. As in past years, a global decline in TB incidence rates has been reported. The European WHO Region has reported stronger annual declines than those observed for the world as a whole (5.2% vs. 1.8%). However, this decline does not meet the target necessary to achieve the milestones of the End TB Strategy by 2025 [4].

In Germany, the incidence rate between 2002 and 2012 fell continuously. After 2012, there was a period of stagnation, followed by a rise of 27.3% in a comparison of incidence rates between 2014 and 2015. Only since 2017 have the incidence rates started to fall again, with 5495 newly reported cases of TB, or a decline of 7.7% [5].

Healthcare workers are at particular risk of infection by *Mycobacterium tuberculosis*, the pathogen responsible for TB, through patient contact [6,7,8,9]. Both fingerprinting studies and systematic analyses in conventional studies on infection risk have shown that many TB cases among healthcare workers are occupational in nature. In the Hamburg fingerprinting study, 80% of the TB onset cases among healthcare workers for which an infection chain was found were occupational in nature. In a comparable Dutch study, this value was 43% [10,11].

TB is the second most common work-related infectious disease among German healthcare workers [12]. Due to the elevated risk involved, healthcare workers (HW) are regularly screened for TB [13,14,15]. For TB to be recognized as an occupational disease (OD), the risk of infection at the workplace, relative to the general risk of infection among the general population, is crucial [11,16,17,18]. Despite the decline in TB incidence rates among the general population, there has been a rise in the number of occupational TB cases in recent years in Germany. Changes in risk perception and better diagnoses as a result of the interferon-gamma release assay (IGRA) may explain this trend over time [9]. According to the WHO, around 10% of those infected with TB suffer from a manifestation of TB. Half of them experienced TB in the first two years after infection, while the other half experienced it over the later course of their lives [4,19]. For an infection to progress to TB, there are various risk factors, including young age, malnutrition, excessive alcohol intake, and immunodeficiency [20]. This is why it is not clear whether the WHO rule also applies to healthcare workers.

The purpose of this study is to use routine data of the German Social Accident Insurance (DGUV) to study trends in the frequency of TB as an occupational disease. It was determined as comprehensively as possible within which sector of Germany TB cases occur, and possible differences in time trends between private and public sector were investigated. Furthermore, the time between infection and recognition of TB as an occupational disease was investigated and described.

## 2. Materials and Methods

This study is an analysis of annual cross-sectional routine data from the German Social Accident Insurance from 2002 to 2017. Every officially recognized case of tuberculosis as occupational disease in Germany is documented by the DGUV.

### 2.1. Data Sources

Statutory accident insurance is one pillar of Germany’s social security system. As a result of mergers of accident insurances since 2002, only 33 of 56 carriers remained under the roof of the German Social Accident Insurance (Deutsche Gesetzliche Unfallversicherung, DGUV) in 2017. Employees, as well as apprentices (trainees), are subject to social insurance against work-related and commuting accidents and against occupational diseases. The DGUV is divided into the statutory accident insurance institutions for the private sector (Berufsgenossenschaften (BGs)), and the public sector accident insurers (PSAI). There are nine trade-specific accident insurance associations covering the private sector, with the Statutory Accident Insurance of the Health and Welfare Services (Berufsgenossenschaft für Gesundheitsdienst und Wohlfahrtspflege, BGW) being responsible for non-governmental healthcare and welfare institutions. In 2017, BGW insured about 7.4 million persons, while the other BGs insured 46.4 million, and PSAI insured about 9.8 million persons [21].

The analyses were performed using routine data of the DGUV. For the analysis, the cases that were recognized as occupational diseases (OD) with OD No. 3101 (transmission of occupational infectious diseases from person to person) and OD No. 3102 (transmission of occupational infectious diseases from animal to human) in relation to TB were included.

### 2.2. Variables

The analysis was based on the occupational disease routine database (BK-DOK) of the DGUV. The criterion for inclusion was a reportable suspicion of an occupational disease under OD No. 3101 or OD No. 3102. Only recognized tuberculosis cases in the reporting years 2002 to 2017 were included. The following information was collected about the cases selected according to the criteria: year of recognition, insurance carrier, latency between the start of potential exposure, and recognition of the insurance case, as well as occupational activity at the time of infection risk. It was not possible to differentiate between a latent TB infection and active TB cases, based on the data available.

### 2.3. Ethical Approval

No decision of the Ethics Committee is required for this investigation of claims data. In accordance with the Professional Code for Physicians in Hamburg (Art. 15, 1., Status of 10.03.2014) and the Chamber Legislation for Medical Professions in the Federal State of Hamburg (HmbKGH), it is only necessary to obtain advice on questions of professional ethics and professional conduct from an Ethics Committee if data that can be traced to a particular individual is used in a research project. Likewise, the DGUV did not stratify their data for small samples.

### 2.4. Patient and Public Involvement

This was a secondary data analysis, and therefore, there was no direct patient or public involvement.

### 2.5. Statistical Analyses

A descriptive analysis of the DGUV data was first performed. For the purposes of data protection requirements, and in order to ensure that individual persons remained unidentifiable, it was not possible to differentiate between genders, for example, in relation to the latency of the insurance case. An aggregated presentation of various insurance carriers was necessary for the same reason at certain points. OD No. 3102 is only presented as a summary over the entire study period, for reasons of data protection law.

Group differences of categorical variables were evaluated using Fisher’s exact test, and corresponding odds ratios (OR) with 95% confidence intervals (CI) were computed. To examine case number increases of the insurance carriers over time, a general linear model (GLM) with insurance carrier, year, and their interaction terms as fixed effects was fitted to log-transformed data [22,23]. SPSS^®^ Statistics Version 22 [24] and the software package R [25] were used for statistical presentation and analysis. The significance level was set at 5%.

## 3. Results

**Figure 1:**
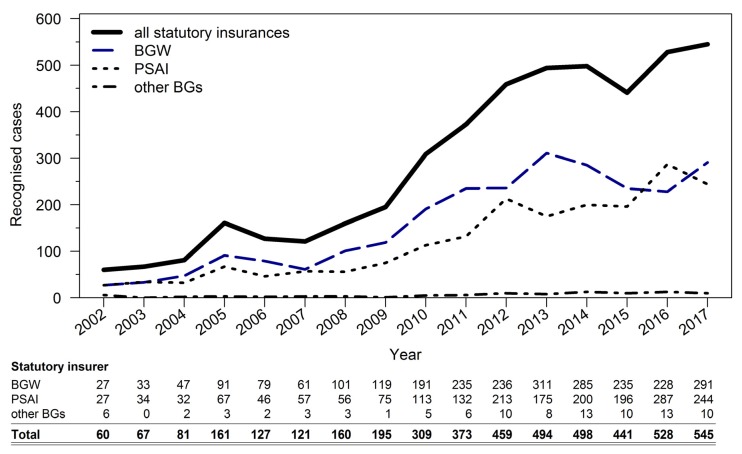
Breakdown by insurance carrier of recognized occupational tuberculosis (TB) cases over the years (only occupational disease (OD) No. 3101).

4653 TB cases were recognized as occupational diseases in the overall period. In 2002, 60 TB cases were recognized as occupational diseases with transmission from person to person. The number of recognized cases more than doubled to 161 until 2005. After stagnating at this level until 2009, there was another tripling of recognized cases until 2013. Between 2013 and 2017, there was a near-stagnation observable with a mild upward trend to a level of around 500 recognized cases per year (see Figure 1). A difference in the relative increase in case numbers over the years was not demonstrable between the three insurance carrier groups (*p* = 0.31).

Regarding occupational groups, the nursing profession (including geriatric nursing) represented the largest group in the DGUV database, with over 2700 recognized cases. The second largest group, with 683 cases, was that of other healthcare workers, followed by physicians with 674 cases. These three groups together account for 88.5% of the recognized tuberculosis cases (see Table 1).

A review of the distribution of recognized OD tuberculosis cases over the years by insurance carrier reveals that case figures of BGW and PSAI were almost equal in 2017 (244 vs. 291 cases). Reported together, they accounted for over 500 recognized cases in 2017. The other accident insurances had 0 to 13 confirmed TB cases per year. At 34, the number of recognized TB cases where the disease was transmitted from an animal (OD No. 3102) was low in the sixteen-year study period (see Table 2).

**Figure 2:**
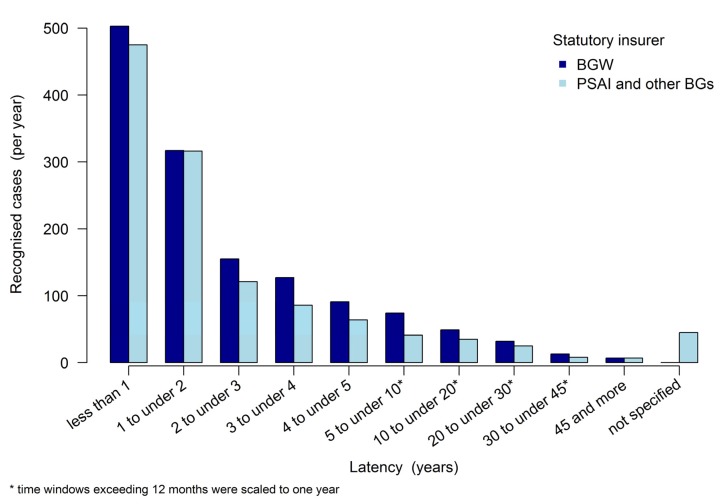
Latency between the potential exposure and the year of recognition as an insurance case (i.e., TB recognised as OD No. 3101 between 2002 and 2017).

The TB recognition latency, i.e., the time between the start of potential exposure and approval as an insurance case or recognition as an occupational disease (“latency period”), is regressive (see Figure 2). The highest recognition figures were reported within the first year (*n* = 978). In the second year, the number of recognized cases was 633, while in the third year it was 276, with a downward trend. BGW and the other insurance carriers do not vary visually in terms of development over the various periods.

Noting the cut-off of two years, the following arose for the years 2002–2017: with a maximum latency of two years, BGW reported 820 (32%) of the 2570 total recognized OD No. 3101 cases. Accordingly, 68% of the recognized cases exhibited a latency of more than two years. Other insurance carriers (i.e., PSAI and other BGs) saw a latency of no more than two years with 791 (39%) of 2004 recognized occupational disease cases. However, both latent and active TB can be accepted as an OD. In this group consisting of “PSAI and other BGs”, no latency was specified for 45 recognized occupational disease cases (see Table 3).

The differences in proportional values for BGW and PSAI and other BGs with a maximum latency of two years are statistically significant, with an odds ratio of 1.39, 95% CI: (1.23 to 1.58), and *p* < 0.001. Within these two groups, the division into long latencies (>2 years) and short latencies (≤2 years) differed significantly in relation to an equal distribution into short and long latencies (both *p* < 0.001).

## 4. Discussion

The novelty in our paper is the description of time trends of TB as occupational disease in the different sectors of the German economy. The analysis of the data revealed 4619 recognized OD No. 3101 TB cases in the sixteen-year study period. There has been a significant increase over the years. This increase may be explained by a number of causal factors, such as significant improvements in diagnostic instruments in terms of sensitivity and specificity, due to the IGRA test, particularly in detecting latent tuberculosis infection (LTBI) [15,26,27,28].

In our analysis, we found an increasing trend of tuberculosis as OD. In the years 2012 to 2016, the figures of newly reported cases of TB in Germany rose as well. The number increased from 4219 in 2012 to 5926 registered cases in 2016, corresponding to an incidence increase from 5.2 in 2012 to 7.2 cases per 100,000 of the population in 2016 [5].

In contrast, the routine data of BGW showed a downward trend in other blood-borne infectious diseases, such as hepatitis, in recent years. Between 2013 and 2017, the number of cases of hepatitis B fell from 46 to 28, and the number of cases of hepatitis C fell from 52 to 29 per year [29].

After 2003, TB cases where the infection time was more than two years in the past may also be recognized as occupational diseases. This allows for infection risks dating further back to be taken into consideration. Therefore, since 2003, reports of suspected cases relate both to events that had recently occurred and events preceding the report by several decades.

The results from the IGRA test generally do not allow for differentiation between an LTBI and an acute TB ailment. An IGRA value above the threshold of 0.35 IU/mL only allows for a conclusion of past contact with tuberculosis bacteria; in its own right, it provides no information about the activity status of the disease. It is also not possible to conclude, on the basis of the IGRA test, whether the infection occurred recently or further in the past. This can only be done by assessing the potential time of exposure. Another point to be considered relates to the latency in recognizing an occupational disease; recognition of an LTBI is generally more likely to involve a shorter latency than a disease manifestation. In LTBI cases, the IGRA test is performed by a competent works physician eight weeks after contact with an infectious patient. This means that the test result directly follows the contact, and because the cases reported by us are only recognized occupational disease cases, the test produced a positive result. On the other hand, the onset of a TB infection as active TB may not occur until years after contact with an infectious patient, in which case symptoms of the disease are usually the trigger for an X-ray examination and possibly a sputum sample. However, TB symptoms are initially non-specific. The time that passes until an X-ray examination occurs must also be taken into consideration alongside the time it takes for the onset of the disease. Both times added together generally result in a higher latency. Among the workers in the DGUV database presented here, it was then determined where contact with an infectious person might have occurred. Contact dating back years prior was evaluated as a (possible) trigger event. Because the cases reported by us are recognized occupational diseases, the contagion was deemed likely to be related to the performance of professional activities, which resulted in recognition of the disease as an occupational disease.

When considering the distribution of recognized occupational diseases across the various insurance carriers, it seems that an increase in recognition figures would be a consistent consequence of taking events further in the past into account. This is exhibited with all insurance carriers; a difference in the increases was not demonstrable among the studied insurance carriers.

In a comparison of occupational groups, healthcare workers exhibited especially high case figures, and the share of recognized OD No. 3101 TB cases in these professional groups was 88.5%. Evidently, due to their frequent contact with patients and clients, health and welfare workers are more at risk of infection than most other workers. The elevated risk of contagion with TB was also reflected in the Hamburg fingerprinting study, where 80% of TB cases in healthcare workers with a known transmission route were caused by an infection acquired in the course of professional activity [10].

## 5. Conclusions

Even though TB incidence in the German population is low, TB in health and welfare workers remains an important issue in both the private and public health and welfare sector in Germany. This is partly due to the long latency period between potential exposure to infectious patients or materials and the recognition of the LTBI or active TB as OD.

## Figures and Tables

**Table 1:** Distribution of recognized OD No. 3101 TB cases in the years 2002–2017, by occupation.

Occupation	Number (%)
Nursing (including geriatric)	2729 (59.1%)
Physicians	674 (14.6%)
Other healthcare employees	683 (14.8%)
Social welfare professions	128 (2.8%)
Administrative/office	98 (2.1%)
Education	32 (0.7%)
Other *	275 (5.9%)
Total	4619 (100%)

* Note regarding “Other”: the occupations listed under this heading could not be categorized, were unknown, or were grouped together for reasons relating to data protection law.

**Table 2:** Distribution of recognized OD No. 3102 TB cases in the years 2002–2017: statutory accident insurance institutions for the private sector (Berufsgenossenschaften, BGs, including the Statutory Accident Insurance of the Health and Welfare Services—Berufsgenossenschaft für Gesundheitsdienst und Wohlfahrtspflege, BGW) vs. public sector accident insurers (PSAI).

Insurance Carrier	Number (%)
Private sector (BGW and other BGs)	20 (58.9%)
Public sector (PSAI)	14 (41.1%)
Total	34 (100%)

**Table 3:** Dichotomized latency of recognized OD No. 3101 TB cases in the years 2002–2017: BGW vs. PSAI and other BGs.

Insurance Carrier	Latency		Total
	≤2 years (%)	>2 years (%)	Number (%)
BGW	820 (31.9%)	1750 (68.1%)	2570 (100%)
PSAI and other BGs*	791 (39.5%)	1213 (60.5%)	2004 (100%)
Total	1611 (35.2%)	2963 (64.8%)	4574 (100%)

* In the group “PSAI and other BGs”, no latency was specified for 45 recognized occupational disease cases.
